# Supplementary material for: Genome-wide identification and characterization of NHL gene family in response to alkaline stress, ABA and MEJA treatments in wild soybean (Glycine soja)
Source: PeerJ. 2022 Dec 2;10:e14451. doi: 10.7717/peerj.14451 (PMC9744164; doi:10.7717/peerj.14451)
Supplement: Supplemental Information 9 [file peerj-10-14451-s009.docx]

**Table S1. Gene-specific primers used in this study.**

| Gene name | Primer sequence (5’-3’) |  |
| --- | --- | --- |
| *GsNHL4* | Forward: GCTCTTTATGGTGGTTCGGTT | |
|  | Reverse: TCGGGGGAGATAGTTGTTGTT | |
| *GsNHL6* | Forward: CGCCGCTACGACCAATACA | |
|  | Reverse: GCTCCTCGGGACGGAAAAC | |
| *GsNHL7* | Forward: ACCCCAACAAAAAACTCAGCA | |
|  | Reverse: CGATCTCGTACACCCCACC | |
| *GsNHL8* | Forward: ACTATCCAAACAACAACACCCTT | |
|  | Reverse: ATCAACACCTTCTGCCCCG | |
| *GsNHL9* | Forward: ATTACTATACCAACAACAACACCCTT | |
|  | Reverse: AACTTGCGAACCTGACATCC | |
| *GsNHL11* | Forward: AAAATCGGAGTCTACTACGACCG | |
|  | Reverse: CAAAAGTTATGAAAGCAGGGC | |
| *GsNHL12* | Forward: GCTGGCGTTGAACGAGAGG | |
|  | Reverse: CGCAGACGATAGTGACAGAGATG | |
| *GsNHL29* | Forward: TCCGCCAATACACAAAGAGC | |
|  | Reverse: GAAGTCACCAAGCCTAAACCTAA | |
| *GsNHL44* | Forward: CCGATAACCCTAACGACAAGA | |
|  | Reverse: CTAACGGTCAACGGCACTCT | |
| *GsNHL45* | Forward: TCACTGCCAGAAACCCCAA | |
|  | Reverse: GGCTGCTTCACCCTCAAAT | |
| *GsNHL47* | Forward: CAGTGGTAGTTGTAGGCATTGTAGT | |
|  | Reverse: GATGGTGAGTTGGGTTTGGA | |
| *GsNHL51* | Forward: CGAAAACCTCCTACCACCG | |
|  | Reverse: GAGCCAGAACACGAACCCG | |
| *GsGAPDH* | Forward: GACTGGTATGGCATTCCGTGT | |
|  | Reverse: GCCCTCTGATTCCTCCTTGA | |
