# Supplementary material for: Genome-wide identification and characterization of NHL gene family in response to alkaline stress, ABA and MEJA treatments in wild soybean (Glycine soja)
Source: PeerJ. 2022 Dec 2;10:e14451. doi: 10.7717/peerj.14451 (PMC9744164; doi:10.7717/peerj.14451)
Supplement: Supplemental Information 10 [file peerj-10-14451-s010.docx]

**Table S2. The information of putative cis-acting elements in *NHL* family genes promoters from wild soybean.**

| Name | *cis*-acting elements | Description |  |
| --- | --- | --- | --- |
| MeJA | TGACG/CGTCA | *cis*-acting regulatory element involved in the MeJA-responsiveness | |
| ABRE | ACGTG/GACACGTACGT/TACGTGTC/CACGTG/AACCCGG/GCAACGTGTC | *cis*-acting element involved in the abscisic acid responsiveness | |
| GA | TCTGTTG/CCTTTTG/AAACAGA | *cis*-acting element involved in the gibberellin responsiveness | |
| SA | CCATCTTTTT/TCAGAAGAGG/GAGAAGAATA | *cis*-acting element involved in salicylic acid responsiveness | |
| Auxin | TGACGTAA/AACGAC | *cis*-acting element involved in Auxin responsiveness | |
| MBS | CAACTG | MYB binding site involved in drought-inducibility | |
| TC-rich | GTTTTCTTAC/ATTCTCTAAC/ATTTTCTTCA | *cis*-acting element involved in defense and stress responsiveness | |
| LTR | CCGAAA | *cis*-acting element involved in low temperature responsiveness | |
