# Supplementary figures and images for: Genome-wide identification and characterization of NHL gene family in response to alkaline stress, ABA and MEJA treatments in wild soybean (Glycine soja)
Source: PeerJ. 2022 Dec 2;10:e14451. doi: 10.7717/peerj.14451 (PMC9744164; doi:10.7717/peerj.14451)

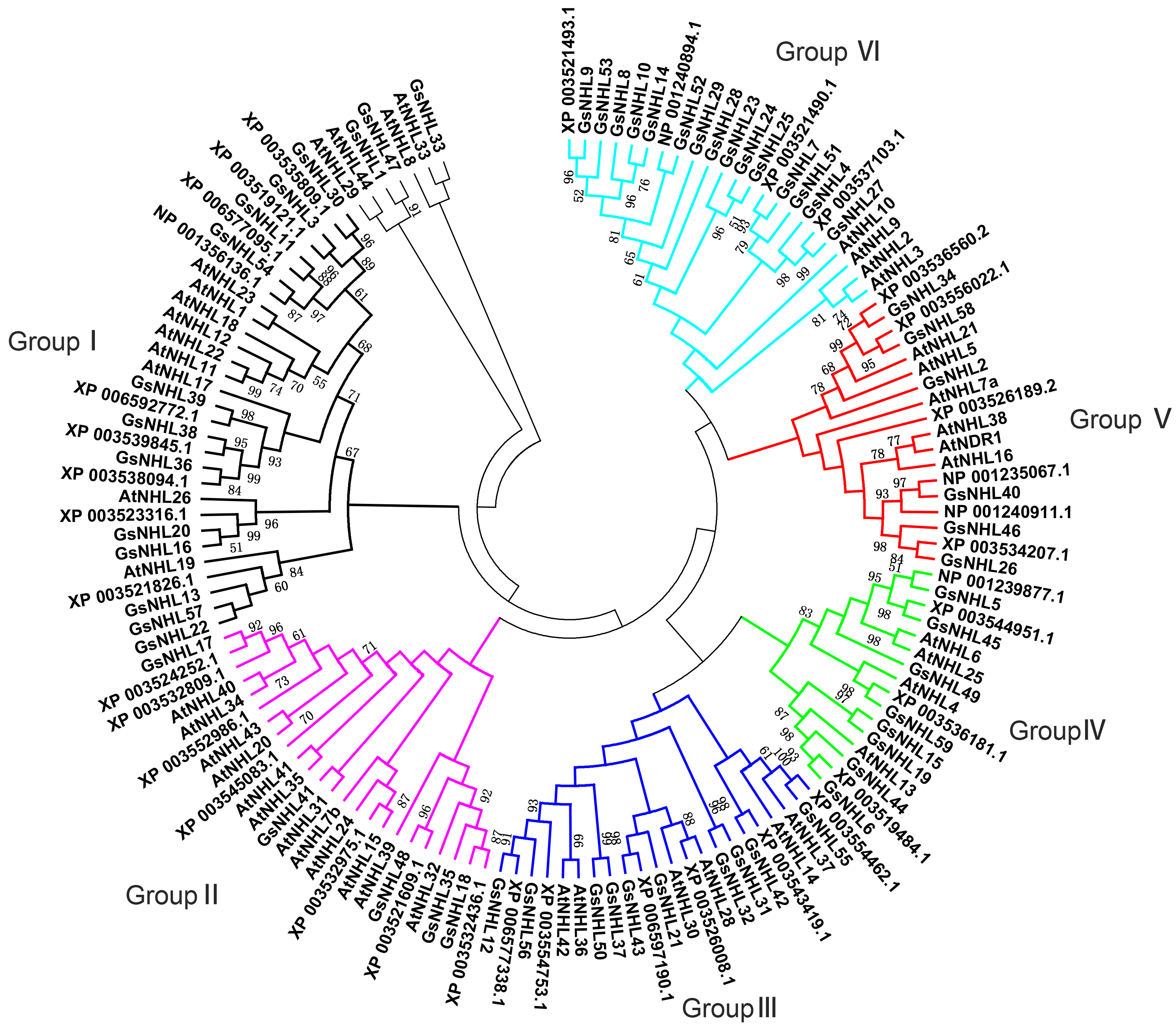

Supplement: Supplemental Information 11 — The maximum-likelihood (ML) phylogenetic tree was constructed based on 1000 replications for each branch. The groups are shown in different colors [file peerj-10-14451-s011.png]

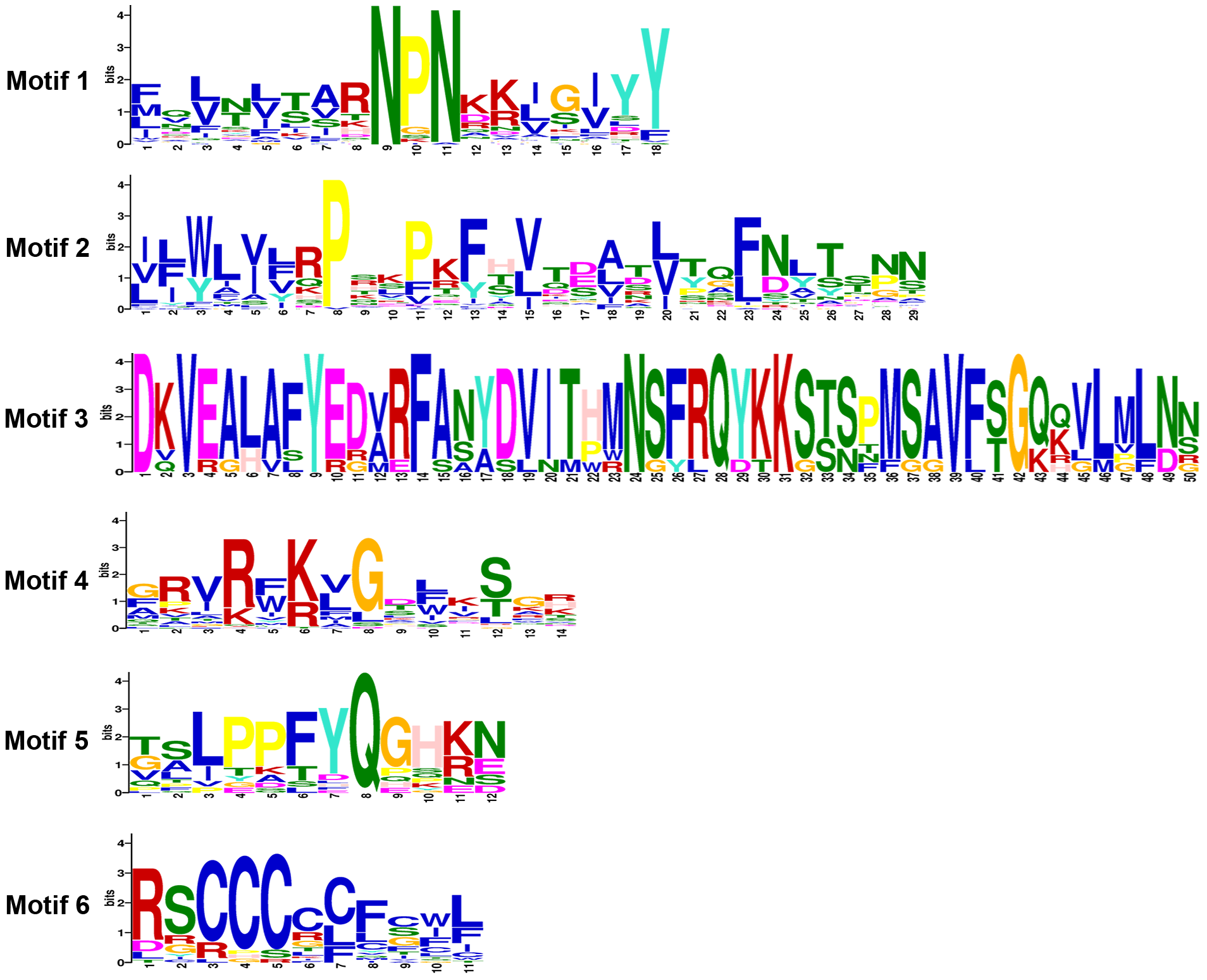

Supplement: Supplemental Information 12 — All motifs were identified by MEME using the complete amino acid sequences of NHL family genes. [file peerj-10-14451-s012.png]
